# Endothelial, platelet, and macrophage microparticle levels do not change acutely following transcatheter aortic valve replacement

**DOI:** 10.1186/s12952-016-0051-2

**Published:** 2016-04-11

**Authors:** Julio F. Marchini, Ayumi Aurea Miyakawa, Flavio Tarasoutchi, José Eduardo Krieger, Pedro Lemos, Kevin Croce

**Affiliations:** Laboratory of Genetics and Molecular Cardiology, Heart Institute, University of São Paulo Medical School, São Paulo, SP Brazil; Valvular Heart Disease Unit, University of São Paulo Medical School, São Paulo, Brazil; Hemodynamics and Interventional Cardiology Service, Heart Institute, University of São Paulo Medical School, São Paulo, SP Brazil; Cardiovascular Division, Department of Medicine, Brigham and Women’s Hospital, Harvard Medical School, Boston, MA USA

**Keywords:** Severe aortic stenosis, Microparticles, Nanoparticle-tracking analysis, Flow cytometry

## Abstract

**Background:**

Patients with severe aortic stenosis have increased levels of prothrombotic and proinflammatory microparticles (MP), and MPs actively regulate pathological processes that lead to atherothrombotic cardiovascular events. Shear stress is a validated stimulus of MP production, and abnormal shear stress in aortic stenosis increases MP release in *ex-vivo* studies. We hypothesized that in patients with severe aortic stenosis, percutaneous replacement of the aortic valve (TAVR) would reduce abnormal shear stress and would decrease levels of circulating MPs.

**Findings:**

The experimental protocol utilized flow cytometry (FC) and nanoparticle tracking analysis (NTA) to quantify circulating plasma MP levels in aortic stenosis patients at baseline and 5 days after TAVR. The baseline and 5 day MP counts measured by FC were 6.10⋅10^5^ ± 1.21⋅10^5^ MP/μL and 5.74⋅10^5^ ± 9.54⋅10^4^ MP/μL, respectively (*p* = 0.91). The baseline and 5 day MP counts measured by NTA were 9.29⋅10^13^ ± 1.66⋅10^13^ MP/μL and 3.95⋅10^14^ ± 3.11⋅10^14^ MP/μL, respectively (*p* = 0.91). When MPs were stratified by cell source, there was no difference in pre/post TAVR endothelial, platelet, or leukocyte MP levels.

**Conclusion:**

Levels of circulating MPs do not change acutely following TAVR therapy for aortic stenosis.

Trial registered at clinicaltrials.gov NCT02193035 on July 11, 2014.

**Electronic supplementary material:**

The online version of this article (doi:10.1186/s12952-016-0051-2) contains supplementary material, which is available to authorized users.

## Findings

### Background

Microparticles (MPs) are submicron membrane fragments that impair endothelial function, promote thrombosis, and increase the risk of cardiovascular (CV) events [1–5]. Endothelial cells, platelets, and macrophages release MPs in response to inflammatory activation and apoptotic signaling [6–8]. Endothelial and platelet MPs circulate at elevated levels in patients with severe aortic stenosis [9]. Shear stress is a validated stimulus of MP production. Abnormal shear stress in aortic stenosis increases MP release in *ex-vivo* studies [7, 9]. We hypothesized that in patients with severe aortic stenosis, percutaneous replacement of the aortic valve (TAVR) would reduce abnormal shear stress and would decrease levels of circulating plasma MPs.

### Methods

**Fig. 1:**
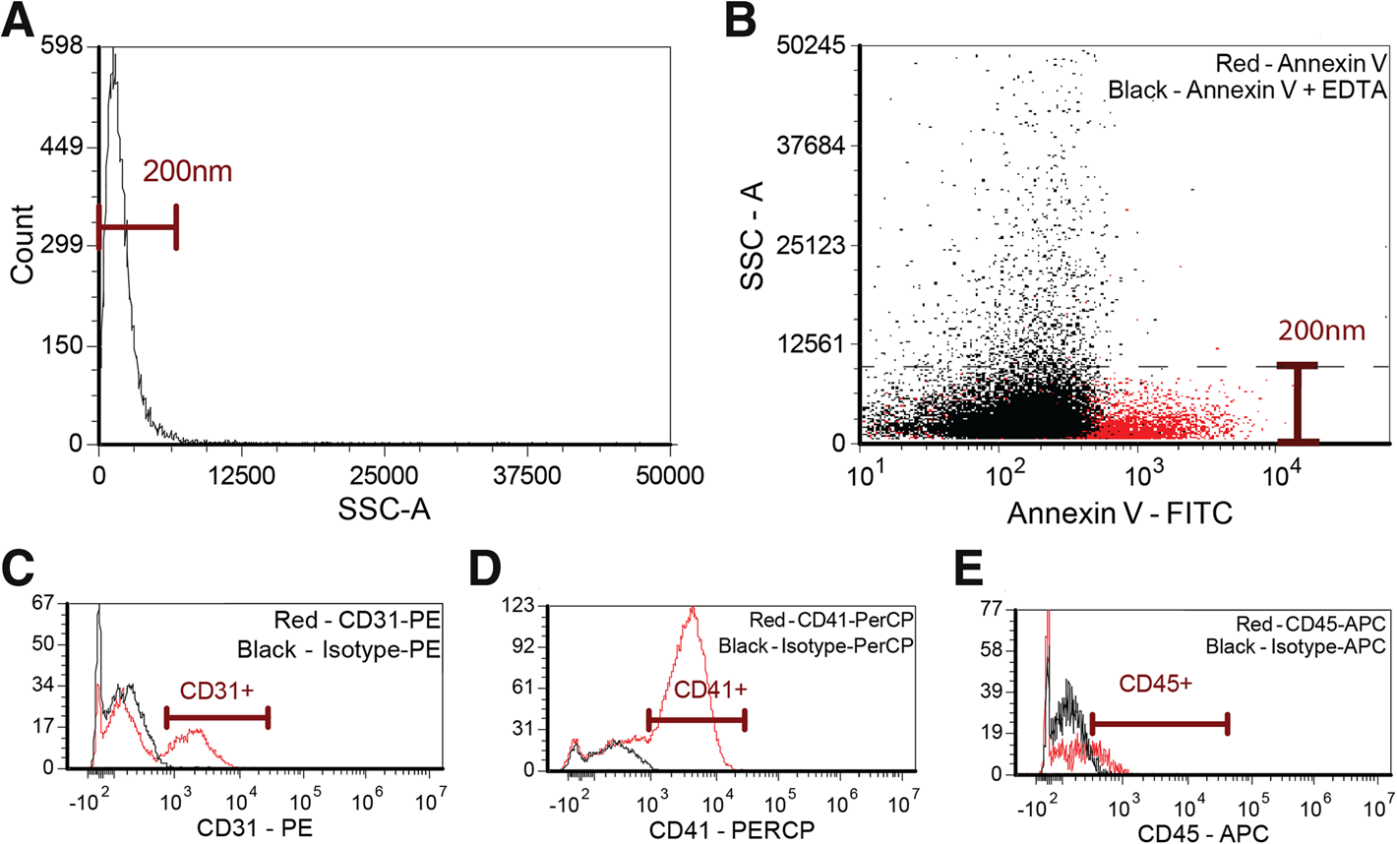
Microparticle Flow Cytometry Quantification Method. **a** Side scatter profile of 200 nm silica size reference beads. **b** Annexin-V stained microparticles (MP) (*red*) and Annexin-V stained MPs treated with EDTA (*black*). The SSC threshold derived from the reference beads is represented by a dashed line (**c**-**e**) Representative flow cytometry data of cell specific antibodies to subtype and quantify endothelial, platelet, and macrophage MPs. We defined endothelial MPs as Annexin V+/CD31+ events, platelet MPs as Annexin V+/CD41+ events, and macrophage MPs as Annexin V+/CD45+ events. Red; specific antibody vs. black; isotype control. SSC; side scatter intensity

This study included patients with severe aortic stenosis selected for TAVR. Pre-specified exclusions are listed in the Additional file 1. The institutional review board approved the protocol (Comissão de Ética para Análise de Projetos de Pesquisa—CAPPESQ / FMUSP #12079). The protocol registration number is NCT02193035 (clinicaltrials.gov). All patients provided informed consent to participate in the study. The experimental protocol used complementary flow cytometry (FC) and nanoparticle-tracking analysis (NTA) to measure MPs. FC identifies MPs based on size and based on MP affinity for Annexin V. Annexin V binds to the prothrombotic lipid, phosphatidylserine, on the outer layer of the MP surface membrane. FC has a limited ability to detect particles that are smaller than 50–100 nm, and thus FC does not accurately quantify ultra-small MPs [10]. Figure 1 shows representative flow cytometry data of 200nM reference sizing beads (Fig. 1a), Annexin-V positive MPs (Fig. 1b), EDTA-treated negative controls (Fig. 1b), and cell specific antibodies to subtype and quantify endothelial, platelet, and macrophage MPs (Fig. 1c-e).

NTA identifies all MPs, including those, which do not have phosphatidylserine on their outer surface. Compared to FC, NTA detects MPs with a lower limit of 50 nm size, and thus NTA has a better sensitivity for ultra-small particles [11]. For this study, MP levels were quantified at baseline and 5 days after TAVR. Detailed methods are available in the Additional file 1.

### Results

We evaluated 15 patients that were enrolled over the course of 12 months and excluded six patients from the study; the reasons for exclusion were fatal stroke (1 patient), fatal cardiogenic shock (1 patient), severe infection (3 patients), and deep venous thrombosis (1 patient). Baseline characteristics of the 9 patients that we analyzed are presented in Table 1.

**Table 1:** Patient and procedure characteristics and outcomes

Characteristics	*N* = 9
Age	84.8 ± 5.1
Male	67 %
Euroscore (Logistic)	23.5 ± 12.5 %
Peripheral artery disease	11 %
End stage renal failure	0
Creatinine (mg/dL)	1.52 ± 0.6
LV-Ao peak gradient	66.4 ± 26.2
LV-Ao mean gradient	43.9 ± 18.2
AVA (cm^2^)	0.7 ± 0.1
Medtronic Corevalve Size	
26	22 %
29	56 %
31	22 %
Outcomes	
Post-TAVR LV-AO peak gradient	13.8 ± 6.4
Death within 30 days	0
Stroke/TIA	0
MI	0
Pacemaker	44 %
Other Complications	11 %

*LV*-*AO* left ventricle—aortic, *AVA* aortic valve area, *TAVR* transcatheter aortic valve replacement, *TIA* transient ischemic attack, *MI* myocardial infarction, *AF* atrial fibrillation. Other complications includes arrhythmias, major bleeding and renal failure

**Fig. 2:**
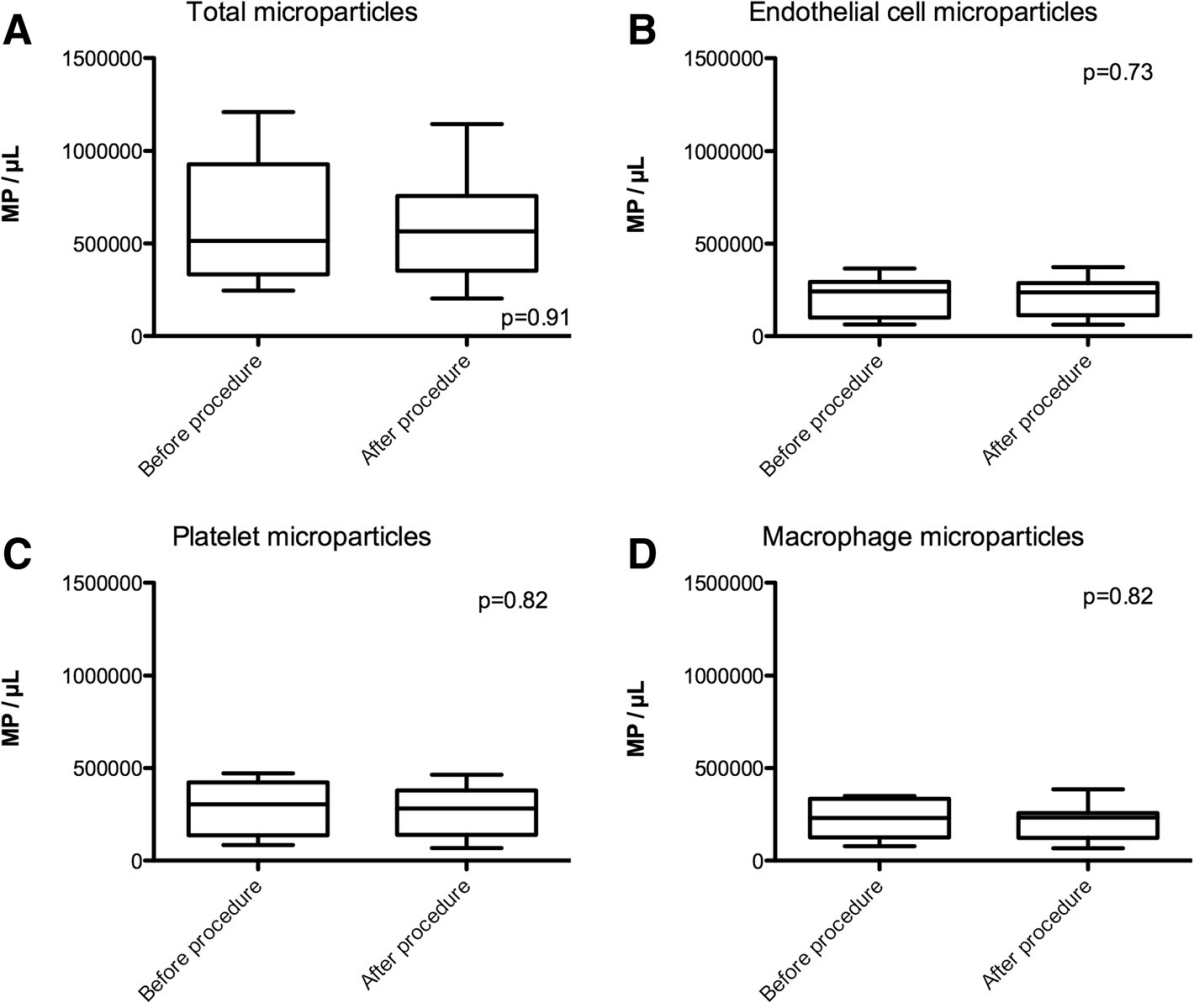
Flow cytometry measurement of microparticle levels before and 5 days after TAVR procedure. **a** Total phosphatidylserine positive microparticles, (**b**) endothelial microparticles, (**c**) platelet microparticles, and (**d**) macrophage microparticles. MP; microparticles

We identified the cell origin of MPs by detecting antibodies directed against endothelial cells, platelets, and leukocytes. The flow cytometry quantification of MPs pre- and 5-day post-TAVR is shown in Fig. 2. There was no difference in total, endothelial cell, platelet, or macrophage MP counts prior to vs. 5 days after TAVR. Total Annexin V positive MPs were 6.10⋅10^5^ ± 1.21⋅10^5^ pre-TAVR vs. 5.74⋅10^5^ ± 9.54⋅10^4^ MP/μL post-TAVR (*p* = 0.91). Endothelial MPs were 2.05⋅10^5^ ± 3.61⋅10^4^ MP/μL pre-TAVR vs. 2.17⋅10^5^ ± 3.38⋅10^4^ MP/μL post-TAVR (*p* = 0.73). Platelet MPs were 2.79⋅10^5^ ± 5.02⋅10^4^ MP/μL pre-TAVR vs. 2.74⋅10^5^ ± 4.41⋅10^4^ MP/μL post-TAVR (*p* = 0.82). Macrophage MPs were 2.18⋅10^5^ ± 3.49⋅10^4^ MP/μL pre-TAVR vs. 2.07⋅10^5^ ± 3.23⋅10^4^ MP/μL post-TAVR (*p* = 0.82).

**Fig. 3:**
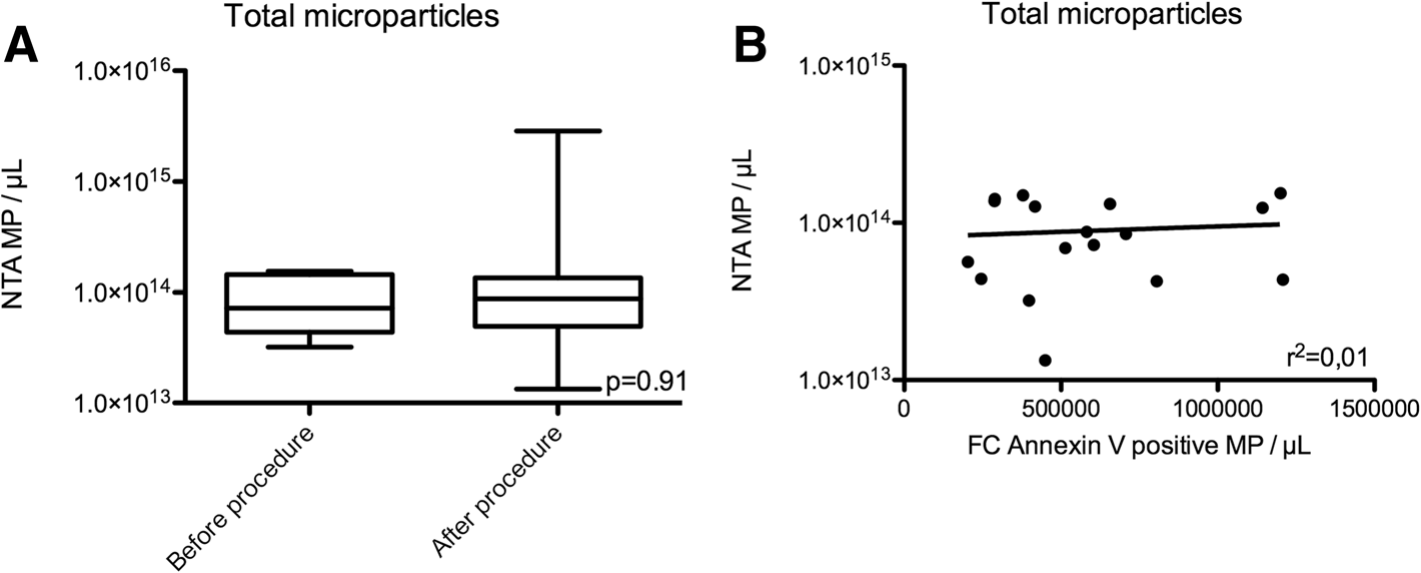
Nanoparticle tracking analysis measurement of microparticle levels before and 5 days after TAVR procedure. **a** Total microparticle levels. **b** Flow cytometry Annexin V positive microparticle events vs. nanoparticle-tracking analysis microparticle events with linear regression comparison between the two MP quantification methods. MP: microparticles; NTA: nanoparticle-tracking analysis

Similar to the FC quantification, there was no difference in NTA-measured MP counts pre vs. 5 days post-TAVR (9.29⋅10^13^ ± 1.66⋅10^13^ MP/μL pre-TAVR vs. 3.95⋅10^14^ ± 3.18⋅10^14^ MP/μL post-TAVR, *p* = 0.91, Fig. 3a). In our data set, there was no correlation between MP levels measured by FC vs. NTA (Fig. 3b, *r*^2^ = 0.01).

Previous investigations have demonstrated that there is a 40 % drop in platelet count following TAVR with a significant incidence of new thrombocytopenia [12]. In our study, the average baseline platelet count was 220⋅10^9^/L ± 90⋅10^9^/L pre-TAVR, and 183⋅10^9^/L ± 78⋅10^9^/L post-TAVR (*p* = 0.0093). Platelet counts fell by an average of 17 %, and 44 % of patients developed thrombocytopenia.

### Discussion

Although the sample size could be larger, our initial results predict a futile effort to show a difference in microparticle level pre TAVR vs. 5 days post TAVR. Previous reports demonstrated a 16.8 % decrease in microparticles 90 days post-TAVR [13]. We therefore simulated adding new cases where all the additional cases have a 20 % decrease in microparticle counts. To calculate the likelihood of this scenario we used the distribution of our original sample. In it, we observed a 10 % average increase in flow cytometry (FC) microparticle counts from pre-TAVR to post-TAVR with a standard deviation of 60 %. Assuming this is a normal distribution, a 20 % decrease corresponds to half a standard deviation less than the mean. The area of the normal distribution below half a standard deviation is 30.9 %, which means that 30.9 % of patients would have at least a 20 % decrease in microparticles. Therefore, the likelihood of a scenario where all six patients present a 20 % decrease in microparticles is 30.9 % to the power of 6, which is 0.09 %.

Next, we did the same modeling for the nanoparticle tracking analysis (NTA). The estimated likelihood of a scenario where all the additional patients would have a decrease of 20 % in microparticle levels is 0.03 %. Thus, there is less than 0.1 % chance that increasing the sample size would have an impact on the mean change in microparticle levels for both methods.

Moreover, if we expanded the sample size to 15 patients, and patients number 10 through 15 each had a microparticle level that was 20 % less than their pre-TAVR level, the p value for the difference would still be non-significant for both methods (modeled p value for FC: 0.51 and NTA: 0.13). Therefore, we conclude that there is no justification to expand the study, because doing so would not alter the results or conclusions.

In our investigations, patients with severe aortic stenosis had higher levels of MPs compared to published reports of MP levels in healthy patients. One study that used similar flow cytometry methods found 3.14⋅10^5^ MP/μL (IQR 2.27⋅10^5^–4.45⋅10^5^ MP/μL) total MPs [14, 15]. Meanwhile, protocols using NTA methodology report MP levels of 1–5⋅10^12^ MP/μL in healthy controls subjects [11, 16]. Notably, our finding that FC MP quantification varied by an order of magnitude compared to NTA MP quantification corroborates similar findings from another group [17]. An explanation for the difference in MP counts between the two methods is that unlike FC, NTA quantifies large, small, and ultra-small MPs, as well as both phosphatidylserine positive and negative MPs [17].

Similar to previous reports, we observed marked thrombocytopenia following the TAVR procedure [12]. Platelet consumption has the potential to increase platelet activation and increase associated production of platelet MPs. Despite the potential link between platelet count and MP levels, we saw no relationship between these two parameters in our study (correlation between platelet count, absolute platelet decrease and FC-measured MP, *r*^2^ = 0.02 and *r*^2^ = 0.16, respectively; correlation between platelet count, absolute platelet decrease and NTA-measured MP, *r*^2^ = 0.08 and *r*^2^ = 0.06; NTA, respectively).

This current clinical study demonstrates that circulating MP levels do not change acutely 5 days following TAVR. Although the number of patients enrolled in this study was small, based on the observed means and standard deviation, an increase in sample size would not change the overall conclusions. When interpreting the negative results of this study, it is important to consider the following points: (1) the half-life of circulating vascular MPs is unknown, and if MPs circulate for several days, our brief 5 day follow-up window might prevent detection of changes in MP levels post TAVR, (2) thrombotic or inflammatory processes may be playing a greater role than shear stress in promoting MP elevation in patients with aortic stenosis, (3) the acute stress of the TAVR procedure might promote MP production—potentially negating the reduction in MP generation achieved by replacing the stenotic valve, and (4) the 5 day follow-up time that we chose might be too short to enable a resetting of homeostatic systems that activate MP release. Of note, a recent investigation demonstrated that circulating MP levels do decrease 90 days following TAVR [13]. The later time point of 90 days may provide adequate time for recovery from the TAVR procedure and/or resetting of homeostatic systems that promote MP production [13, 18].

### Conclusions

Our data found increased MP levels in patients with severe aortic stenosis in comparison to reports of healthy patients. We did not observe a reduction of MP levels in a short follow-up of TAVR. MPs, which have prothrombotic and proinflammatory effects, imply an increased risk of CV events for patients with severe aortic stenosis that persists acutely after TAVR treatment. Targetting MP generation or effects could reduce TAVR periprocedural CV events.

## Additional file

Additional file 1: Detailed description of the methods. (DOCX 25 kb)

## Abbreviations

FC: flow cytometry
MP: microparticle
TAVR: transcatheter aortic valve replacement
NTA: nanoparticle-tracking analysis
