# Supplementary material for: Endothelial, platelet, and macrophage microparticle levels do not change acutely following transcatheter aortic valve replacement
Source: J Negat Results Biomed. 2016 Apr 11;15:7. doi: 10.1186/s12952-016-0051-2 (PMC4827212; doi:10.1186/s12952-016-0051-2)
Supplement: Additional file 1: — Detailed description of the methods. (DOCX 25 kb) [file 12952_2016_51_MOESM1_ESM.docx]

**Additional file**

# **Endothelial, platelet, and macrophage microparticle levels do not change acutely following transcatheter aortic valve replacement.**

Julio F. Marchini*^ac^, Ayumi Aurea Miyakawa^a^, Flavio Tarasoutchi^b^, José Eduardo Krieger^a^, Pedro Lemos^c^, Kevin Croce^d^

## Methods

Pre-specified exclusion criteria were chosen to avoid conditions that might stimulate microparticle production independent of aortic stenosis. The exclusion criteria were: severe aortic insufficiency, deep vein thrombosis, sepsis, or active malignancy. We also excluded patients that had pre or post TAVR procedure hemodynamic instability, stroke, myocardial infarction, or death.

The protocol collected venous blood into 3.2% citrate tubes at the start of the TAVR procedure (before heparin administration) and on day 5 days (± 1 day) after the TAVR procedure. Microparticle processing occurred within 3 hours of phlebotomy. The protocol for isolating microparticles (MPs) consisted of two sequential centrifugation steps; 15 minutes at 1500G and 2 minutes at 13000G [2]. After collecting the upper two-thirds of the platelet poor plasma microparticle fraction, we stored the samples at −80**°**C to allow for batched analysis. The precipitated platelet fraction (pellet) served as a positive control for the FC and NTA quantification because freezing platelets releases large amounts of MPs. All samples were analyzed after a single thaw.

We performed statistical analysis with Graphpad Prism 5 using non-parametric paired tests to compare microparticle counts (Wilcoxon matched pairs test). Linear regression analysis enabled assessment of the correlation between the FC and NTA methods.

The FC protocol quantified microparticle levels using a FACS Accuri C6 flow cytometer (Becton Dickinson, Franklin Lake, NJ). Table S1 list the source of Annexin V and cell antibodies used in this study (Annexin V-FITC, CD31-PE, CD41a-PerCP-Cy5.5, and CD45-APC). We used reference sizing beads to establish microparticle size and to set side scatter (SSC) thresholds (fluorescent green silica beads 200nm – #141114-10 – Corpuscular, Cold Spring, NY) (Figure 1)[3, 4]. In the FC analysis, we defined MPs as Annexin V-positive particles less than 200nM, which had an Annexin-V fluorescence value greater than the 99th percentile of the EDTA-treated negative controls (Figure 1). Annexin V binds to the prothrombotic phospholipid phosphatidylserine (PS), which is a component microparticle surface membrane. Annexin-V-PS binding is calcium dependent, and calcium chelation with 20mM EDTA abolished Annexin V binding to the microparticles (Figure 1). Flow cytometry experiments employed cell specific antibodies to subtype and quantify endothelial, platelet, and macrophage MPs. We defined endothelial MPs as Annexin V^+^/CD31^+^ events, platelet MPs as Annexin V^+^/CD41^+^ events, and macrophage MPs as Annexin V^+^/CD45^+^ events. Fluorescent counting beads (Flow-Count Fluorosphere, Beckman Coulter, Brea, CA) normalized FC flow variation between samples and enabled precise quantification of FC counts. We used FCS Express Flow Cytometry Software, version 4.0 (DeNovo Software, Los Angeles, CA) to analyze the FC data.

The nanoparticle-tracking analysis protocol utilized a Nanosight NS300 (Malvern, UK) and software version 3.1 to quantify the MPs [5]. We defined MPs as particles which had a size less than 200nm, and measured each sample three times for 30 seconds in order to accumulate a minimum of 1000 valid tracking events.

## List of abbreviations

FC = flow cytometry; MP = microparticle; TAVR = transcatheter aortic valve replacement; PS = phosphatidylserine.

**Table S1. Annexin V and Fluorescent Antibodies used for flow cytometry**.

| **Protein / Antibody** | **Catalog number** | **Supplier** |
| --- | --- | --- |
| **Annexin V - FITC** | BMS306FI/100 | eBioscience |
| **CD31-PE** | 555446 | BD Pharmingen |
| **CD41a PerCP-Cy5.5** | 340931 | BD Pharmingen |
| **CD45 APC** | 340943 | BD Pharmingen |
| **PE Isotype Control** | 349043 | BD Pharmingen |
| **PerCP-Cy5.5 Isotype Control** | 552834 | BD Pharmingen |
| **APC Isotype Control** | 340442 | BD Pharmingen |

## References

1. **Drug-induced nephrotoxicity: an international symposium. Antwerp, Belgium, September 27-28, 1985.** *Am J Kidney Dis* 1986, **8:**283-383.

2. Jy W, Horstman LL, Jimenez JJ, Ahn YS, Biro E, Nieuwland R, Sturk A, Dignat-George F, Sabatier F, Camoin-Jau L, et al: **Measuring circulating cell-derived microparticles.** *J Thromb Haemost* 2004, **2:**1842-1851.

3. Tzur A, Moore JK, Jorgensen P, Shapiro HM, Kirschner MW: **Optimizing optical flow cytometry for cell volume-based sorting and analysis.** *PLoS One* 2011, **6:**e16053.

4. van der Pol E, Coumans F, Varga Z, Krumrey M, Nieuwland R: **Innovation in detection of microparticles and exosomes.** *J Thromb Haemost* 2013, **11 Suppl 1:**36-45.

5. Hutcheson JD, Goettsch C, Pham T, Iwashita M, Aikawa M, Singh SA, Aikawa E: **Enrichment of calcifying extracellular vesicles using density-based ultracentrifugation protocol.** *J Extracell Vesicles* 2014, **3:**25129.
